# Possibility of determining the degree of adhesion of the lymph node to the pulmonary artery preoperatively

**DOI:** 10.1186/s13019-015-0310-1

**Published:** 2015-07-25

**Authors:** Hidetaka Uramoto, Satoshi Nozu, Yuki Nakajima, Hiroyasu Kinoshita

**Affiliations:** 1 Divisions of Thoracic Surgery, Saitama Cancer Center, 780 Komuro, Ina, Kita-adachi-gun, Saitama 362-0806 Japan; 2 Division of Diagnostic Radiology, Saitama Cancer Center, 780 Komuro, Ina, Kita-adachi-gun, Saitama 362-0806 Japan

**Keywords:** Lung cancer, Degree of adhesion of the lymph nodes, Inflammatory lymph nodes, Calcified lymph node, Surgical resection

## Abstract

**Background:**

To make a preoperative determination of the degree of adhesion of the lymph node (LN) to the pulmonary artery (PA) in patients with lung cancer.

**Methods:**

We investigated clinical parameters, including sex, age, smoking, stage, histology and surgical procedure, and performed an image analysis using CT scanning.

**Results:**

The data for sex, age, smoking, stage, histology and the surgical procedure were identical between the “adhesion” and “no adhesion” groups. However, three of the five analyzable cases in the adhesion group clearly showed the disappearance of the fat plane on minimum intensity projection (minIP) computed tomography (CT). In particular, sites on more than three slices demonstrated the disappearance of the fat plane. On the other hand, five of the eight analyzable cases in the no adhesion group showed no disappearance of the fat plane. Therefore, one central slice adequately reflected the fat plane in the cases in the no adhesion group.

**Conclusions:**

These findings suggest that it is necessary to obtain a careful diagnosis of the extent of attachment of the LN to the PA using modern diagnostic imaging in order to preoperatively assess the degree of adhesion of the LN to adjacent structures.

## Background

**Fig. 1:**
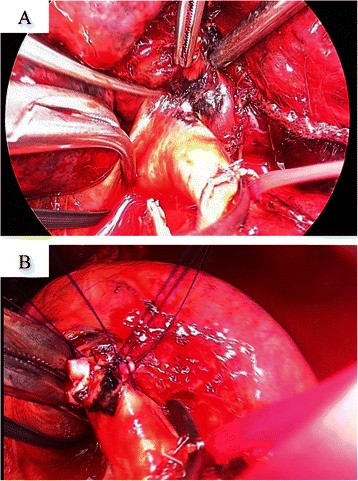
**a** Operative findings showing adhesion between the LN and PA, which is hazardous (Case 3 in Table 1). **b** Findings of anthracosis on the PA surface after PA reconstruction following clamping of the main PA and peripheral PA for LND

Lung surgery was first developed to treat inflammatory lung tumors, such as those associated with tuberculosis. Therefore, the surgical terminology regarding inflammatory, infiltrating and calcified LN arose from such procedures. Recently, the number of operations for lung cancer has been increasing due to the rise in the incidence of disease as a result of the aging of the population and new developments in diagnostic imaging [1]. However, calcification of the LN, even in cases without extra capsular extension of lymph node swelling, usually occurs as a sequel to old granulomatous disease and is a possible cause of adhesion [2]. On the other hand, video-assisted thoracoscopic surgery (VATS) lobectomy has come into widespread use for the treatment of early lung cancer, with the advantages of minimal invasiveness [1], thus allowing the surgeon to treat cases in which the LN cannot be removed intraoperatively. Furthermore, safety and quality are required for proper medical care. Troublingly, most LNs are located in the vicinity of the left PA [3], especially in cases of reverse A3, which is a hazardous location (Fig. 1a). To complicate matters, physicians sometimes have difficulty in estimating the potential for peeling from the PA after cutting the superior pulmonary vein. Hence, this decision to exfoliate the LN from the PA is made intraoperatively. When combining the resection of the PA, the surgeon must save the main trunk of the PA and perform a PA plasty (Fig. 1b). As a consequence, the operation is time-consuming [4]. Furthermore, histopathological analyses sometimes show the absence of cancer cells in the LN [5]. Therefore, it is critical to determine the degree of adhesion of the LN preoperatively. However, few available reports on this issue exist. The purpose of the current study was to describe the clinical factors and chest CT findings and evaluate the possibility of determining the degree of adhesion of the LN to the PA preoperatively in patients with lung cancer.

### Clinical experience

The preoperative investigations included chest radiographs and high-resolution CT scans of the thorax. The preoperative clinical stage was determined based on the findings of CT, positron emission tomography (PET) and brain magnetic resonance imaging (MRI). One hundred and seventy patients underwent surgical resection between June 2014 and December 2014 at the Saitama Cancer Center, Japan. Among these subjects, 108 patients with primary lung cancer underwent surgery. There were four N1 disease patients with adhesion of the LN to the PA, and eight N1 disease patients without adhesion (three patients who underwent surgery after induction chemoradiotherapy were excluded). The patients’ records, including their clinical data, preoperative examination results, details of the surgeries and histopathological findings, were also reviewed. Former smokers were defined as those who had quit smoking at least three years before the time of surgery. Clinical factors, including CT findings, were compared directly between the patients. The protocol for postoperative management and follow-up has been described in a previous report [6].

### Technique

#### CT scanning and image analysis

Whole-lung CT scans were obtained with a 128-detector row CT scanner (SOMATOM definition flash, Siemens) using the following technique: 0.6 mm collimation, 0.5 s rotation time, 5 mm thick reconstructions, pitch ratio of table travel per rotation to total beam width) of 120 kVp in the radiation dose generated by the CARE Dose4D software. All images were reviewed with 3 mega-pixel monochrome LCD monitors (Eizo Nanao Corporation) with standard lung (window width, 1500 HU; window level, −500 HU) and mediastinal (window width, 300 HU; window level, 40 HU) window settings. Composite images of sites of the LN bordering with the PA were made for made for direction of tangent 1 mm by SYNAPSE VINCENT® (Fujifilm Medical Co., Tokyo, Japan). MinIP images, not maximum intensity projection (MIP) images, were used to improve visualization of the fat plane at a distance of 3 mm. The resultant images were transferred to a picture archiving and communication system (PACS) (SYNAPSE-Fujifilm) for the image analysis. One board-certified thoracic radiologist, who was blinded to the operative findings and perioperative outcomes, retrospectively evaluated 1-mm-thick axial and sagittal images in consensus in a direction perpendicular to the PA.

### Operative procedure

All patients underwent general anesthesia using a double lumen endotracheal tube for single-lung ventilation. The operation was performed in the lateral decubitus position using a utility incision and two 12-mm ports. Open thoracotomy was selected in preference to VATS in patients in potentially life-threatening situations depending on the assessment of the operating surgeon. The definition of adhesion of the LN was a degree of adhesion to the adjacent structures beyond the realm of possibility of being hidden (Fig. 1a). When the adhesion of the LN was recognized, the clamping of the main PA and peripheral PA was performed for lymph node dissection (LND).

## Results

**Fig. 2:**
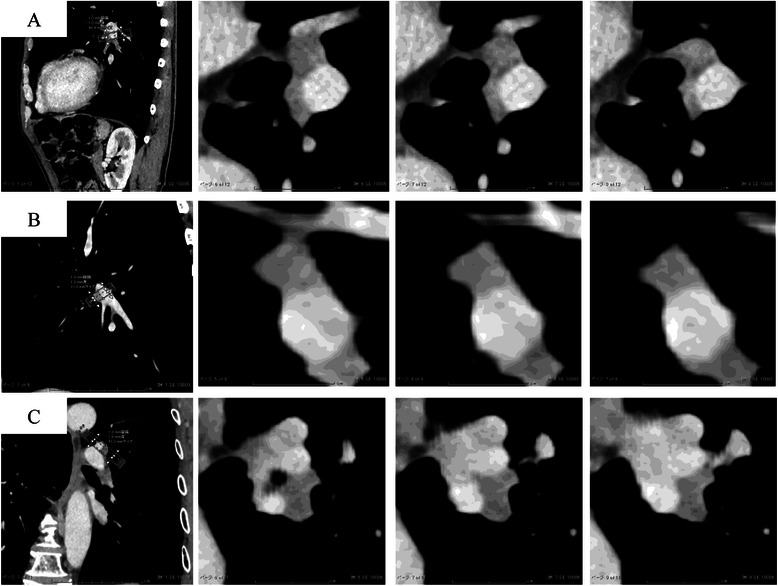
**a** Sagittal view (*left periphery*) and sections for the LN direction tangent to the PA (case 1), **b** Case 2, **c** Coronal section views (*left periphery*) and sections for the LN direction tangent to the PA (case 3)

**Fig. 3:**
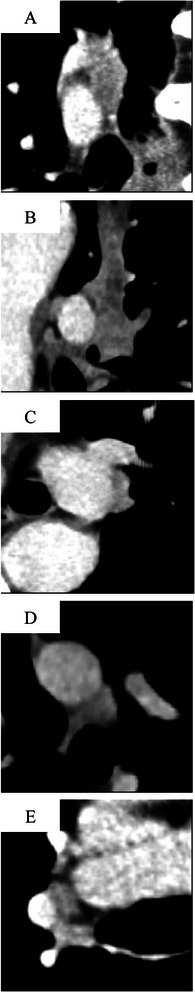
One central slice shows the fat plane in series. **a** case 1, **b** 2, **c** 3, **d** 4, and **e** 5 in Table 2

All patients were Japanese. A summary of the details of all of the cases is given in Tables 1 and 2. The data for sex, age, smoking, stage, histology and surgical procedure were identical between the adhesion and no adhesion groups. However, three of the four analyzable cases judged to have adhesion of the LN to the PA clearly showed the disappearance of the fat plane on minIP CT. In particular, sites on more than three slices demonstrated disappearance of the fat plane (Fig. 2). On the other hand, five of the eight analyzable cases in the no adhesion group showed no disappearance of the fat plane. Therefore, one central slice adequately reflected the fat plane in the cases without adhesion of the LN (Fig. 3). There were no cases of postoperative mortality (30-day mortality and hospital mortality after chest surgery), and all patients were alive at the time of the analysis.

**Table 1:** Characteristics of the cases with adhesion of the LN to the PA

Case	Sex	Age	Smoking	c-Stage^a^	s-Stage^b^	p-Stage^c^	Histology^d^	Surgical procedure^e^	CT finding
1	M	63	Former	IA	IA	IA	AD	LLL + LND	disappearance of fat plane
2	F	71	never	IA	IB	IA	AD	RML + LND	disappearance of fat plane
3	M	69	current	IB	IB	IB	SQ	LUL + LND with PA reconstruction	disappearance of fat plane
4	M	69	current	IIA	IB	IA	SQ	LUL + LND	nd^f^

^a^clinical stage, ^b^surgical stage, ^c^pathological stage, ^d^*SQ* squamous cell carcinoma, *AD* adenocarcinoma, ^e^*LLL* left lower lobectomy, *LND* lymph node dissection, *RML* right middle lobectomy, *LUL* left upper lobectomy, ^f^not investigated due to plain CT

**Table 2:** Characteristics of the cases without adhesion of the LN to the PA

Case	Sex	Age	Smoking	c-Stage	s-Stage	p-Stage	Histology	Surgical procedure^a^	CT finding
1	M	79	Former	IIIA	IV	IV	SQ	RLL + LND	Existence of fat plane
2	M	79	Former	IIA	IIB	IIA	SQ	RUL + LND	Existence of fat plane
3	M	69	Former	IB	IIA	IB	AD	LUL + LND	Existence of fat plane
4	M	63	current	IB	IIA	IIA	SQ	LUL + LND	Existence of fat plane
5	M	76	current	IIA	IIA	IIA	SQ	RLL + LND	Existence of fat plane
6	M	65	current	IIB	IIIA	IIIA	AD	RUL + LND	nd^b^
7	M	55	current	IIB	IIIA	IIA	SQ	RUL + LND	nd^b^
8	M	77	current	IIB	IIA	IIB	AD	RLL + LND	nd^b^

^a^*RLL* right lower lobectomy, *RUL* right upper lobectomy, ^b^not investigated due to a lack of analyzable data

## Discussion

To the best of our knowledge, there have been no previous reports concerning the methods for determining the degree of adhesion of the LN to the PA preoperatively in patients with primary lung cancer. This study included one expected and one novel finding. First, the data for sex, age, smoking, stage, histology and surgical procedure were identical between the no adhesion and adhesion groups. Initially, we predicted that a male sex, smoking habit and histology of squamous cell carcinoma may affect the degree of adhesion of the LN. However, our findings did not support this hypothesis. Nevertheless, the number of patients in our series was very small. Therefore, future studies must be designed to confirm our outcomes in analyses of larger numbers of cases.

Second, among the cases with adhesion of the LN, sites on more than three slices showed the disappearance of the fat plane on minIP CT. However, one central slice adequately demonstrated the fat plane in the cases without adhesion of the LN. We constructed the data from minIP CT, not MIP, images in order to emphasize the fat layer to the extent possible [7]. However, the comparison showed no significant differences between the minIP CT and MIP findings. Importantly, our series did not include calcified LNs. Furthermore, routine systematic lymph node dissection (LND) is widely performed, as calcification of the LNs does not preclude the presence of malignancy [5], and such dissection may affect the perioperative outcomes [8]. Hence, further detailed examinations are required, and it is necessary to accurately determine the degree of adhesion of the LN in order to improve the safety and quality of the operation.

There are several limitations that must be taken into account when considering the present findings. These limitations include the retrospective nature of the study and the fact that it was carried out at a single institution. There were also imbalances in the patient characteristics that could be excluded due to the small number of patients without using statistical procedures, and the presence of selection bias must be acknowledged. Moreover, the interpretations of the radiograms may have been subjective. Nevertheless, the current results highlight an important issue, as surgery should be performed safely, and the development of diagnostic imaging may provide useful information for improving surgical quality. This strategy is feasible and may thus contribute to improving the results of surgical treatment.

### Consent

The informed consent was obtained from the patients for the use of their data in the analyses described herein.

## Abbreviations

LN: Lymph node
PA: Pulmonary artery
minIP: Minimum intensity projection
CT: Computed tomography
VATS: Video-assisted thoracoscopic surgery
PET: Positron emission tomography
MRI: Brain magnetic resonance imaging
MIP: Maximum intensity projection
LND: Lymph node dissection
